# Multiple Infections, Nutrient Deficiencies, and Inflammation as Determinants of Anemia and Iron Status during Pregnancy: The MINDI Cohort

**DOI:** 10.3390/nu16111748

**Published:** 2024-06-02

**Authors:** Doris González-Fernández, Elizabeta Nemeth, Emérita del Carmen Pons, Delfina Rueda, Odalis T. Sinisterra, Enrique Murillo, Veena Sangkhae, Lisa Starr, Marilyn E. Scott, Kristine G. Koski

**Affiliations:** 1 School of Human Nutrition, Macdonald Campus, McGill University, Ste-Anne de Bellevue, QC H9X 3V9, Canada; doris.gonzalez-fernandez@mail.mcgill.ca; 2 Center for Iron Disorders, David Geffen School of Medicine, University of California, Los Angeles, CA 90089, USA; enemeth@mednet.ucla.edu (E.N.); vsangkhae@mednet.ucla.edu (V.S.); 3 Department of Nutritional Health, Panamanian Ministry of Health, Panama City 7098, Panama; emeritapons@gmail.com; 4 Comarca Ngäbe-Buglé Health Region, Panamanian Ministry of Health, San Félix, Panama; delfinaminsa@gmail.com; 5 Panamá Norte Health Region, Panamanian Ministry of Health, Panama City 7104, Panama; odalisin@gmail.com; 6 Department of Biochemistry, University of Panama, Panama City 7096, Panama; emurillo29@hotmail.com; 7 Institute of Parasitology, Macdonald Campus, McGill University, Ste-Anne de Bellevue, QC H9X 3V9, Canada; lisa.starr@mcgill.ca (L.S.); marilyn.scott@mcgill.ca (M.E.S.)

**Keywords:** anemia, pregnancy, indigenous, hepcidin, undernutrition, iron, infections

## Abstract

In pregnant women with multiple infections, nutrient deficiencies, and inflammation (MINDI), the study of anemia and iron status is limited. For this cross-sectional study (*n* = 213 Panamanian indigenous women), we investigated if hemoglobin, anemia (Hb < 110 g/L), ferritin, serum iron, serum transferrin receptor, and hepcidin were associated with (1) maternal nutritional status and supplementation practices, (2) biomarkers of inflammation, and (3) presence/absence of infections. Hierarchical generalized linear and logistic regression models and dominance analyses identified the relative importance of these predictors. Anemia (38%), which was likely underestimated due to low plasma volume (95%), was associated with lower ferritin, vitamin A, and weight-for-height, suggesting anemia of undernutrition. Inflammation was not associated with Hb or anemia; nevertheless, higher CRP was associated with increased odds of low serum iron and higher ferritin and hepcidin, indicating iron restriction due to inflammation. The length of iron supplementation did not enter models for anemia or iron indicators, but a multiple nutrient supplement was associated with higher ferritin and hepcidin. Moreover, iron supplementation was associated with higher odds of vaginal trichomoniasis but lower odds of caries and bacterial vaginosis. The complex pathogenesis of anemia and iron deficiency in MINDI settings may require other interventions beyond iron supplementation.

## 1. Introduction

Anemia during pregnancy affects more than 56 million women worldwide, particularly in low–middle income countries (LMIC) [1,2]. Pregnant women are more prone to developing anemia due to physiological plasma volume expansion during pregnancy and due to increased iron and nutrient demands for placental and fetal growth [3]. Despite important reductions in anemia in women of reproductive age in Latin America, anemia is still a severe public health problem in some countries [4], and regional disparities are common. In Panama, where this study was conducted, the prevalence of anemia in remote areas exceeds 40% [5], in contrast to national estimates of 22.6% [6]. 

Presently, anemia prevention and control programs rely on iron supplementation, as it is understood that about 50% of anemia is due to iron deficiency [7]. However, the contribution of iron deficiency to anemia is lower in populations exposed to inflammation [8], and other micronutrient deficiencies also contribute to anemia [7]. Moreover, the identification of nutritional iron deficiency is challenging in developing settings given the co-existence of multiple factors, particularly the coexistence of nutritional iron deficiency with other nutritional anemias and with iron restriction due to inflammation [9]. Moreover, iron status indicators are themselves modified by other nutritional deficiencies and by inflammation [10]. This may underscore why public health efforts directed towards iron supplementation have not decreased iron deficiency in resource-poor settings [11] and have led to the re-evaluation in the last decade of the concept that anemia is synonymous with iron deficiency, particularly after studies showed that the co-occurrence of infections with micronutrient deficiencies contribute to anemia in developing settings [12].

In addition to these challenges, interpretation of traditional biomarkers of iron status (hemoglobin, ferritin, serum transferrin receptor (sTfR)), and other less frequently used indicators such as serum iron and hepcidin, is complex, especially during pregnancy. Hemoglobin is traditionally used to screen for iron deficiency, but hemoglobin lacks sensitivity to detect early iron deficiency [13]. Hemoglobin normally decreases during pregnancy in what has been called ‘physiologic anemia of pregnancy’ as a result of the discordant increase in plasma volume (50%) and red cell mass (30%) [14]. Currently, the WHO recommends the use of serum ferritin for evaluating the iron status of populations [15] and provides a cut-off for low ferritin during the first trimester but not for the second or third trimesters. Moreover, in non-pregnant populations, a higher ferritin cut-off is recommended in the presence of inflammation, determined by the elevation of acute phase indicators, usually C-reactive protein (CRP) [16]. However, the WHO does not currently provide a cut-off for low ferritin in pregnant women with infections or inflammation [15]. 

A workup for the detection of iron deficiency in clinical practice includes other biomarkers such as sTfR and serum iron. Circulating sTfR primarily derives from erythroid transferrin receptor 1 and reflects the number of erythroid precursors and their cellular iron status; therefore, elevated sTfR is found in iron-deficient erythropoiesis and is an early biomarker of iron depletion [17]. Given that the main contributor to sTfR concentrations is the bone marrow erythroid precursor mass [18], sTfR has been used to assess the response to iron supplementation [19] and the erythropoietic activity during pregnancy [20,21]. Serum iron has been used to determine iron status during pregnancy [22,23] and continues to help in the diagnosis of iron deficiency in some LMIC settings [24,25]. However, because serum iron concentrations decrease in the presence of inflammation even when iron stores may not be depleted [26], serum iron is not recommended as the sole measurement of iron status in individuals or populations [27,28], or during pregnancy [29].

More recent research has introduced hepcidin as a promising biomarker of iron status [30,31]. Healthy pregnancies are characterized by lower hepcidin compared to the nonpregnant state and with a progressively greater decrease in hepcidin as pregnancy advances [32]. Hepcidin is still regulated by iron during pregnancy and positively correlates with serum ferritin, but with a descendent shift in their correlation curves from the first to second trimester, so that hepcidin levels are nearly 10-fold lower in the second trimester over a similar range of ferritin [33,34]. The physiological decrease in hepcidin during pregnancy has been used to assess the safety of administering iron in developing settings [35,36], with higher hepcidin concentrations predicting poor response to iron supplementation [37] as well as increased non-absorbed iron in the intestinal lumen, which can alter the balance of the healthy intestinal microbiome [38]. However, studies to date do not clearly indicate a benefit of using high hepcidin to identify women who will not benefit from iron supplements [39]. Moreover, the lack of cut-offs for hepcidin or the hepcidin/ferritin ratio during pregnancy hampers the diagnostic value of hepcidin [40].

In Panama, iron deficiency is considered the main cause of anemia [41], but the non-response to iron supplementation, particularly in indigenous communities, has been a public health concern. Despite the distribution of iron and multiple nutrient supplementations to pregnant women, anemia has a prevalence > 30% in the Ngäbe-Buglé indigenous community [42]. Our previous work on these pregnant women also revealed low protein status [43], multiple micronutrient deficiencies (folic acid, B12, vitamin A and D), inflammation measured through elevated C-reactive protein (CRP) [44], and high prevalence of oral, skin, urogenital, and intestinal parasitic infections [45]. As iron supplementation may be counter-productive in conditions of infection/inflammation [46], and as there is evidence that anemia and iron status are variably modulated by different infections [47], we postulated that the combination of multiple infections, nutrient deficiencies, and inflammation might contribute to the persistent anemia and differentially impact individual iron status indicators in pregnant women from marginalized communities.

**Figure 1:**
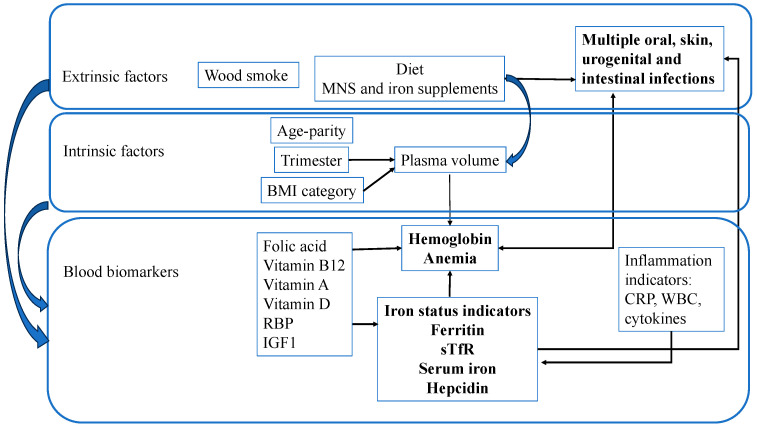
Conceptual framework. BMI: body mass index; CRP: C-reactive protein, IGF1: insulin-like growth factor 1; RBP: retinol-binding protein; sTfR: serum transferrin receptor; WBCs: white blood cells.

We created a conceptual framework (Figure 1) based on the WHO’s rationale for a comprehensive framework for accelerating anemia reduction [48] as a tool for exploring relationships among our MINDI (Multiple Infections, Nutrient Deficiencies and Inflammation) variables. Our framework explored known extrinsic and intrinsic factors and biomarkers associated both with anemia and individual iron status indicators. In our framework, infections were not considered to cause anemia, as malaria was not endemic; analyses showed no association of hookworm infection with anemia, and other oral, skin, urogenital, and intestinal infections are known to not be directly associated with anemia. Our specific objectives were the following: (1) to identify among a set of nutritional and inflammatory biomarkers those that were associated with anemia and individual iron status indicators (hemoglobin, ferritin, serum iron, sTfR, hepcidin) while controlling for relevant maternal factors; (2) to explore if anemia and other iron status indicators were associated with the intake of a multiple nutrient supplement (MNS) or iron supplementation; and (3) to assess whether iron status indicators and iron supplementation were associated with the presence/absence of bacterial, protozoan, and nematode infections.

## 2. Materials and Methods

### 2.1. Recruitment, Ethics, and Questionnaire Data

All subjects gave their informed consent for inclusion before they participated in the study. The study was conducted in accordance with the Declaration of Helsinki, and the protocol was approved by the Ethics Committee of the Gorgas Memorial Institute in Panama (no. 1618/CNBI/ICGES/10), which required as a prerequisite for final approval a signed agreement with indigenous authorities of the Ngäbe-Buglé comarca and with the Panamanian Ministry of Health. Ethics approval was also obtained from the Institutional Review Board of McGill University in Canada (no. A03-M16-10A).

For this cross-sectional study, 213 pregnant women from the Ngäbe-Buglé indigenous territory in western Panama were recruited between August and December 2010. Obstetric history, exposure to wood smoke, diet (recall of times in the past week having eaten animal-source foods, green/leafy vegetables, and red/yellow/orange fruits or vegetables), duration of intake of 1–3 60 mg iron tablets/d, and tbsp/d of a MNS “Nutricrema” were collected, as described previously [43]. At the time of the study, MNS was distributed free of charge to all except overweight/obese pregnant and lactating women, according to the local protocol. This MNS contained per 100 g (equivalent to approx. 6 tbsp/d recommended by MINSA): 350 Kcal, protein (12 g), lipids (12–14 g), vitamin A (250 µg), iron (11.1 mg), calcium (250 mg), vitamins E (10 mg), C (140–280 mg), B1 (0.5 mg), B2 (0.5 mg), B3 (6 mg), B12 (0.9 µg), folic acid (85 µg), zinc (8.0 mg), and copper (400 µg) [49]. MNS was taken as part of the diet of women and children; therefore, the amount per day in tablespoons, but not duration of supplementation during pregnancy was recorded. Folic acid supplementation, although recommended, had shortages of supply, and therefore, its intake was not recorded. 

Maternal weight by height by gestational age was measured following the Panamanian Guidelines for Pregnancy Prenatal Care [50] and classified using Pan American Health Organization charts as underweight, normal, or overweight/obese [51]. Plasma volume was calculated as total blood volume (TBV) × (1 − hematocrit) [52], where TBV was calculated using Nadler’s equation (TBV = 0.3561 × (height in meters)^3^ + 0.03308 × weight in kg + 0.1833) [53]. A low plasma volume was considered if <2 L in the first, <2.6 L in the second, and <2.8 L in the third trimester, according to values <5th centile found by de Hass et al. [54].

The presence and severity of maternal infections in this cohort have been previously described [45]. Briefly, caries and scabies were clinically detected, and vaginal microorganisms (vaginal yeast, bacteria (*Lactobacillus, Bacteroides/Gardnerella, Mobiluncus*, and *Diplococcus*) and *Trichomonas*) and urinary bacteria were detected using microscopy. Diagnosis of bacterial vaginosis was based on a Nugent score 7–10 [55]. Intestinal parasitic infections (*Ascaris*, hookworm, and *Trichuris*) were detected using direct, Kato–Katz, and Flotac techniques in a subsample of 120 women.

### 2.2. Blood Analyses 

Blood samples were collected from all consenting mothers (*n* = 213), as previously described [56]. For this secondary analysis, subsequent analyses of hepcidin and serum iron from biobanked samples were merged with the previous hematological data.

Hematological data included analysis of whole blood that was processed for red blood cell (RBC) indices and for total and differential white blood cells (WBCs) (BC-5500 Mindray Auto Hematology Analyzer). The WHO cut-offs for anemia (hemoglobin (Hb) < 110 g/L) [57] and for low hematocrit (<33%) [58] were used. Microcytosis was defined as mean corpuscular volume (MVC) < 80 fL, and macrocytosis as MCV > 100 fL [59]. Hypochromia was considered if MCHC < 320 g/L [60], and anisocytosis (variation in RBC size) was considered if red blood cell width (RDW) was >46 fL [61].

Iron status indicators including sTfR immunoassay (RAMCO, Stafford, TX, USA), serum iron (spectrophotometry, FERENE^®^-ENDPOINT), ferritin ELISA (MP Biomedicals Irvine, CA, USA), and hepcidin (Intrinsic Hepcidin IDx ELISA kit, Intrinsic LifeSciences, La Jolla, CA, USA) were analyzed. We considered iron deficiency if elevated sTfR (>8.3 mg/L, RAMCO Laboratories, Chennai, India), or low serum iron (<8.9 µmol/L) [57], or low ferritin, for which two different cut-offs were considered: <15 µg/L (as for non-pregnant women) [15]), and <30 µg/L (used by the Panamanian Ministry of Health [62]). We could not apply the WHO cut-off of <70 µg/L for adult populations with infection/inflammation [15], as only 3% of the study population was above this cut-off. We also considered values of sTfR to be low if <3.0 mg/L, indicating decreased erythropoiesis [63].

Folic acid and vitamin B12 (immunoelectro-chemiluminescence, MODULAR E170 Roche Diagnostics, Mannheim, Germany), vitamin A (HPLC), 1–25 OH vitamin D (LIAISON^®^, DiaSorin, direct competitive chemiluminescence immunoassay, San Diego, CA, USA), retinol-binding protein (RBP, Human RBP4-ELISA, MP Biomedicals) and insulin growth factor-1 (IGF-1, Human IGF-1 single plex, Millipore Corporation, Oakville, ON, Canada) were also measured. Nutrient deficiencies were defined as folic acid < 10 nmol/L [64], and vitamins B12 < 150 pmol/L [64], A < 1.05 µmol/L [65], and D < 50 nmol/L [66]. RBP < 30 mg/L was considered as low protein status [67,68], and low IGF-1 as <25th centile by trimester according to ranges in pregnancy in a Chinese population (49.6, 41.1, and 61.7 µg/L in the 1st, 2nd, and 3rd trimesters, respectively) [69].

C-reactive protein (CRP), interleukin (IL)-1β, IL-4, IL-6, IL-10, IL-12, IL-13, IL-17, interferon (INF)-Υ, tumor necrosis factor (TNF)-α, and monocyte chemoattractant protein 1 (MCP-1) were analyzed using LUMINEX^®^ 200 ^TM^ (Luminex Corp., Montreal, QC, Canada) as part of the Human 10-plex Cytokine/Chemokine Magnetic Bead Panel (Cat. HCYTOMAG-60 K; Millipore Corporation, Oakville, ON, Canada), as previously described [44]. Elevated CRP was considered if >5 mg/L [15], as proposed for identifying inflammation in anemia studies [70].

### 2.3. Statistical Analyses

All statistical analyses were performed using STATA 16 (StataCorp, College Station, TX, USA). Maternal extrinsic (wood smoke, diet, and intake of MNS and iron supplements) and intrinsic (age, parity, trimester, weight-for-height category, and plasma volume) factors and blood biomarkers (Hb, ferritin, serum iron, sTfR, hepcidin, and hepcidin/ferritin ratio) were compared by trimester using the Kruskal–Wallis test for continuous variables and the Chi^2^ test for dichotomous variables, and by anemia status (yes, no). Spearman correlations and graphing of scatter plots with fractional polynomial prediction lines were used to explore associations of months of iron supplementation with the 5 anemia/iron indicators. 

Model strategy: Using our conceptual framework, we ran hierarchical generalized linear models (GLM) for ferritin, sTfR, serum iron, and hepcidin, specifying a Gamma distribution and an identity link. We also ran hierarchical linear and logistic regression models for Hb and anemia, respectively. Independent variables were progressively added by groups: extrinsic factors (wood smoke, diet, and intake of MNS and iron supplements), intrinsic factors (age, parity, trimester, maternal weight-for-height category, and plasma volume), biomarkers of nutrition (folic acid, B12, vitamins A and D, RBP, IGF-1), and biomarkers of inflammation (CRP, cytokines). For the Hb and anemia models, iron indicators (ferritin, serum iron, sTfR, hepcidin, and also the hepcidin/ferritin ratio) were included separately as the last step (proximal predictors). Variables entering models with *p* < 0.20 were retained in each step of the hierarchical process. Correlated covariates were avoided in the same model. To further explore associations of hepcidin with other iron indicators, hepcidin was added to the final models and is reported separately. As recommended for iron-related studies during pregnancy [71,72], all models were controlled for trimester.

To avoid the influence of extreme values of some dependent (hemoglobin, ferritin, and hepcidin) and independent variables (vitamin B12, monocytes, eosinophils, cytokines), a maximum of 2% extreme observations detected by the box-plot method were winsorized [73,74]. If two or more collinear covariates entered a model, we chose the one based on research interest, clinical knowledge, and/or relevance. In particular, for assessing maternal nutritional status as a determinant of anemia, we used the binary variable “low plasma volume” in the logistic model for anemia, allowing for the inclusion of the weight-for-height category (collinear with plasma volume as continuous variable). For the linear hemoglobin model, the continuous variable for plasma volume was used, and a separate model including weight-for-height, while excluding plasma volume, was run.

Dominance analysis was used to determine the relative importance of independent predictors to overall model fit statistics [75]. We report complete dominance, which results from comparing all pairs of independent variables to determine the marginal contribution of each predictor to the model [76]. Linear regression models were assessed for regression assumptions (homoskedasticity, normality of residuals’ distribution, non-collinearity, specification, functional form, absence of outliers) [77]. For GLM models, collinearity was assessed using a variance inflation factor < 10 [78], and the discriminative ability of fitted logistic regression models was assessed using the area under the curve (AUC). Finally, we ran logistic regression models to determine if supplements and the presence of individual infections were associated with iron status indicators while controlling for trimester. Additionally, we ran simple GLM regression models for iron status indicators and months on iron as a factor variable, to observe if iron indicators were associated with different durations of supplementation. 

## 3. Results

### 3.1. General Population Characteristics

The prevalence of anemia was 38% (*n* = 81), and the median hemoglobin concentration for the population (*n* = 213) was 112 g/L. Pregnant women also had a low weekly intake of animal-source foods (median 2 servings with IQR of 1–5), yellow/red fruits and vegetables (1; 0–3), and green/leafy vegetables (1; 0–2). Not surprisingly, these low intakes were accompanied by low RBP (26.9%) and low IGF-1 (76.2%), both indicators of inadequate intake of protein and of protein–energy malnutrition. Furthermore, there was a high prevalence of iron deficiency (ferritin < 15 µg/L or serum iron < 8.9 µmol/L or sTfR > 8.3 mg/L) in 72.3%, as well as multiple micronutrient deficiencies, notably vitamins A (41.4%), B12 (85%), and D (64.8%), and folic acid (23.9%). 

Women were provided with a supplement by the Ministry of Health (MoH). At the time of enrollment, 50% reported taking MNS, half the women had taken MNS on the day the questionnaire was administered, but only six women reported taking the recommended 100 g/d equivalent to 6 tbsp/d of MNS in the past week. Most reported over-dilution and sharing MNS with the entire family, with an intake of 1 (11.3%), 2 (21.6%), 3 (12.2%), or 4+ (5.6%) tbsp/d. Underweight women took 2 tbsp/d of MNS/d (range: 0–9) compared with women of normal weight (1 tbsp, range: 0–6) and overweight/obese women (0 tbsp, range: 0–4; *p* = 0.05).

**Figure 2:**
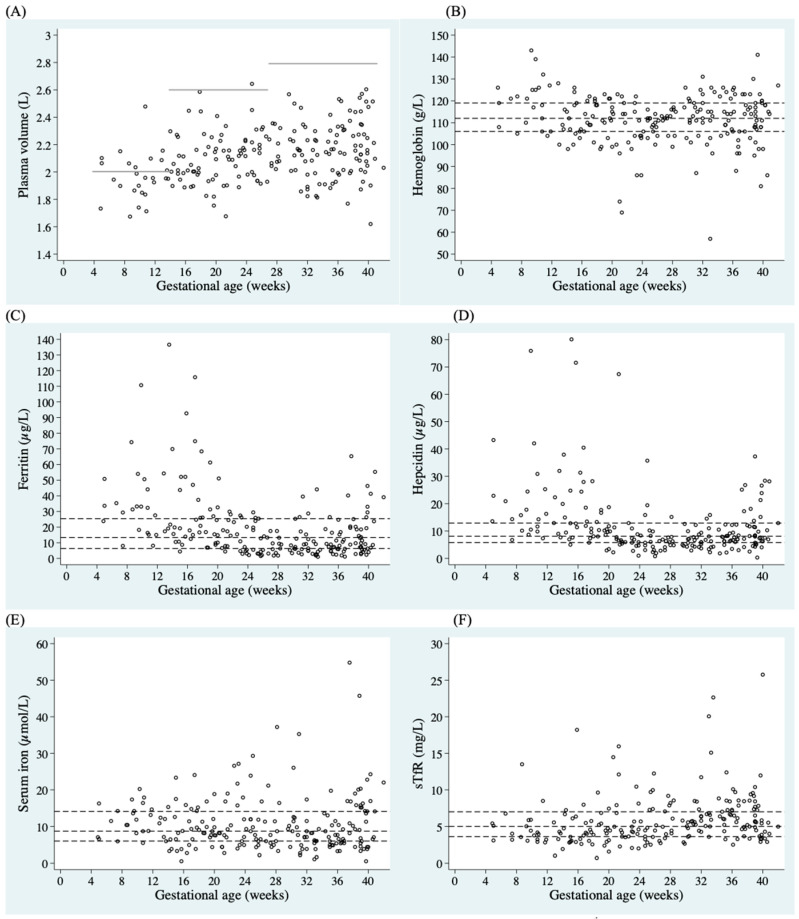
Scatter plots of (**A**) plasma volume, (**B**) hemoglobin, (**C**) ferritin, (**D**) hepcidin, (**E**) serum iron, and (**F**) sTfR, by gestational age (weeks). Dashed lines show specific cut-offs for low plasma volume by trimester, defined as <5th percentile, as reviewed by de Haas et al., 2017 [54]. Dashed horizontal lines show the median and IQR of (**B**) hemoglobin: 112 (106, 119) g/L; (**C**) ferritin: 13 (6.3, 25.4) µg/L; (**D**) hepcidin: 8.1 (5.8, 12.9) µg/L; (**E**) serum iron: 8.7 (6.0, 14.1) µmol/L; and (**F**) sTfR: 5.0 (3.6, 7.0) mg/L.

Scatter plots describing the population distribution for plasma volume and individual iron/erythropoietic indicators (Hb, ferritin, hepcidin, serum iron, and sTfR) by gestational age (weeks) are summarized in Figure 2. Scatter plots revealed that by the second and third trimesters, plasma volume was below the reported fifth centile by trimester for the majority of pregnant women, indicating poor plasma volume expansion in our MINDI cohort as pregnancy progressed. With regards to individual iron status indicators, median and interquartile (25, 75) ranges were plotted. The median concentrations for iron indicators were the following: hemoglobin: 112 (106, 119) g/L, ferritin: 13 (6.3, 25.4) µg/L, serum iron: 8.7 (6.0, 14.1) µmol/L, and sTfR: 5.0 (3.6, 7.0) mg/L. The median concentration of hepcidin was 8.1 (5.8, 12.9) µg/L.

Table 1 describes the differences of iron status indicators and plasma volume and the prevalence of low concentrations in each trimester and highlights both the impact of trimester and worsening iron status during pregnancy in the MINDI cohort. Concentrations of hemoglobin were lower in the second and third trimesters, but the prevalence of anemia did not differ by trimester. Hematocrit was lower in the second compared with the first trimester, but contrary to what was expected, hematocrit in the third trimester did not differ from the first or second trimesters, suggesting a lack of hemodilution. Ferritin concentrations were approximately 50% lower in each successive trimester, which resulted in a high prevalence (70–90%, depending on the cut-off) of inadequate iron stores by the third trimester. In contrast, neither serum iron concentrations nor the prevalence of low serum iron differed across trimesters. sTfR showed higher concentrations in the third trimester, but the prevalence of tissue iron deficiency indicated by high sTfR was not different across trimesters. On the other hand, low sTfR indicative of low erythropoiesis was more prevalent in the second trimester. As expected, hepcidin showed lower values in the second and third trimesters compared with the first trimester, but the hepcidin/ferritin ratio was similar in the first and second trimesters, and this ratio was higher instead of the expected lower ratio in the third trimester (Table 1). Finally, plasma volume was higher in the second and third trimesters compared with the first trimester; however, compared with expected values by trimester, plasma volume was low in most women in the first (61.5%) and second trimesters (98.7%), and in all women in the third trimester.

**Figure 3:**
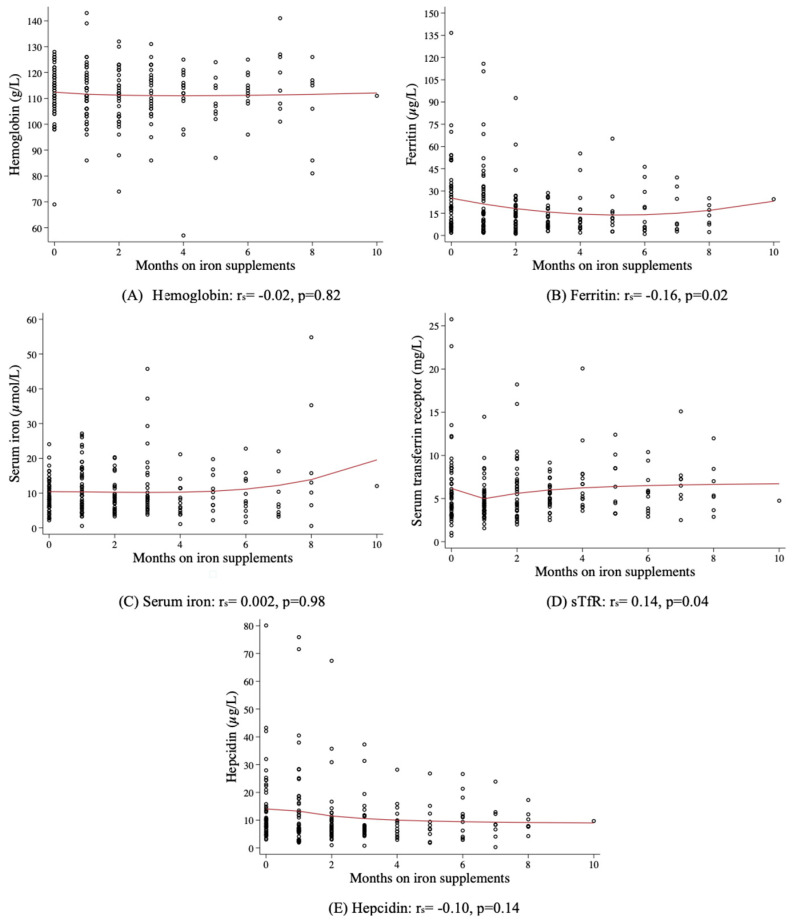
Scatter plots of Hb and other iron status indicators by months taking supplements. Solid red lines denote fractional polynomial regressions. Spearman correlations (r_s_) are shown.

Figure 3 shows scatter plots of iron status indicators by the duration (months) that pregnant women took iron supplements. Most (76.5%) had been taking iron supplements for a median of 2 months (IQR: 1–3), and all anemic women had been taking iron for >3 months. Significant Spearman correlations with low r^2^ were found between ferritin and sTfR with months taking iron, but no correlation was found between Hb, serum iron, or hepcidin with time taking iron, reflecting the lack of efficacy of iron supplementation. GLM regression models of iron status indicators and individual months on iron showed that taking iron supplements for 3 months was inversely associated with ferritin (*n* = 43 women, coef: −11.1 ± 3.6, *p* = 0.002), and taking iron for one month but no longer was inversely associated with sTfR, reflecting only a transient effect on iron status (*n* = 48 women, coef: −1.4 ± 0.6, *p* = 0.029). No other iron status indicators were associated with iron supplementation of any duration.

### 3.2. Comparison of Anemic and Non-Anemic Mothers

Table 2 compares maternal characteristics between anemic and non-anemic mothers. Mothers with anemia had higher plasma volume, but 96.2% of women categorized as non-anemic had low plasma volume, therefore suggesting that the prevalence of anemia may be underestimated. Mothers with anemia also had more previous pregnancies and had mostly low or normal weight for gestational age. However, other indicators of undernutrition (RBP and IGF-1), as well as the intake of iron and MNS, were similar in anemic and non-anemic women. Among micronutrient deficiencies, anemic mothers had a higher prevalence of folic acid deficiency. As expected, ferritin and serum iron were higher in non-anemic mothers, but sTfR concentrations were similar and low erythropoiesis did not differ by anemia status. However, mothers presenting sTfR > 8.3 mg/L were more likely to be anemic. Hepcidin concentrations were similar between anemic and non-anemic mothers, but the hepcidin/ferritin ratio and the proportion of hepcidin/ferritin ratio > 1 were higher in those with anemia, suggesting an inappropriate elevation of hepcidin. Furthermore, despite the high prevalence of deficiencies that can affect RBC size (low ferritin, folate and B12 deficiencies), MCV was within normal range in 84.5% of women but was lower in anemic than in non-anemic women. Microcytic and macrocytic anemia were rare. Hypochromia and anisocytosis were both present, but only hypochromia was more common in anemic compared with non-anemic women.

Table 3 shows infection and inflammation indicators by anemia status. Among the coexisting oral, skin, vaginal, urinary, and intestinal infections (median of 4; IQR: 3–5 infections/mother), the prevalences of bacteriuria and *Mobiluncus* were lower in anemic women. Among inflammation indicators, anemic women had fewer total WBCs (at the expense of lower neutrophils) and lower IL-10. Other WBC subsets, cytokines, and CRP did not differ between anemic and non-anemic women.

### 3.3. GLM Regression Models for Iron Status Indicators

#### 3.3.1. Models for Ferritin and Hepcidin

The models for ferritin (Table 4A) and hepcidin (Table 4B) captured 24.6% and 22.1% of the variability, respectively. Moreover, the set of variables retained in both models was similar. There was a negative association with trimester, which accounted for 53% of the variability in the ferritin model and 43% in the hepcidin model. Vitamin B12 was positively associated with ferritin as second in dominance (coef: 0.06; 95% CI: 0.001, 0.11; *p* = 0.046), but did not show an association with hepcidin (coef: 0.02; −0.001, 0.05; *p* = 0.062). The biomarker of inflammation, CRP, was fifth in dominance for ferritin (coef: 0.38; 95% CI: 0.01, 0.75; *p* = 0.043), and third in dominance for hepcidin (coef: 0.31; 95% CI: 0.12, 0.49; *p* = 0.001), showing positive associations with both ferritin and hepcidin. Among the nutritional status indicators, a higher intake of MNS was positively associated with both ferritin (coef: 1.60, 95% CI: 0.30, 2.91, *p* = 0.016) and hepcidin (coef: 0.87, 95% CI: 0.19, 1.55, *p* = 0.013) (sixth and fifth in dominance, respectively), and higher folic acid concentration was positively associated with hepcidin (coef: 0.14, 95% CI: 0.01, 0.26; *p* = 0.034; fourth in dominance). The number of months taking iron entered the final models for ferritin and hepcidin but had *p*-values > 0.05 in both models.

#### 3.3.2. Models for Serum Iron and sTfR

In contrast to the ferritin and hepcidin models, the models for serum iron (Table 5A) and sTfR (Table 5B) had lower explanatory power (13.6 and 11.7%, respectively) and had different sets of independent variables associated with each outcome. The trimester was not associated with serum iron but was first in dominance in the model for sTfR with a positive association (coef: 1.04, 95% CI: 0.59, 1.55, *p* < 0.0001). Serum iron was negatively associated with two biomarkers of inflammation, monocytes (coef: -14.49; 95% CI: −19.10, −9.87; *p* < 0.0001) and CRP (coef: −0.30; 95% CI: −0.40, −0.20; *p* < 0.0001), which ranked first and third in dominance, respectively. In contrast, none of the inflammation indicators were significantly associated with sTfR, although CRP and eosinophils were retained in the model. Among the nutritional status variables, higher concentrations of folic acid (coef: 0.19; 95% CI: 0.06, 0.31; *p* = 0.004) and vitamin B12 (coef: 0.03; 95% CI: 0.005, 0.05; *p* = 0.016) were positively associated with serum iron (second and fourth in dominance, respectively) but not with sTfR. Interestingly, higher vitamin D concentration was negatively associated with both serum iron (coef −0.06; 95% CI: −0.11, −0.001; *p* = 0.046; sixth in dominance) and sTfR concentrations (coef: −0.03; 95% CI: −0.05, −0.004; *p* = 0.022; second in dominance). Finally, a higher maternal weight-for-height for gestational age was associated with lower sTfR (coef: −0.79, 95% CI: −1.51, −0.07, *p* = 0.030). 

#### 3.3.3. Models for Hemoglobin and Anemia

The logistic regression model for anemia (Table 6A; overall fit 16.5% and AUC = 0.77) and the linear regression model for hemoglobin (Table 6B; overall fit 36% and adjusted R^2^ = 0.33) revealed several parallel results. Ferritin was the main determinant of anemia, with higher concentrations decreasing its odds (OR: 0.96; 95% CI: 0.93, 0.98; *p* = 0.001; first in dominance), and ferritin was second in dominance, predicting higher hemoglobin (*p* < 0.0002; coef: 0.20; 95% CI: 0.12, 0.27). A low plasma volume for gestational age decreased the odds of anemia (OR: 0.13; 95% CI: 0.15, 0.93; *p* = 0.033; sixth in dominance), and higher plasma volume (mL) was first in dominance with a negative association with hemoglobin (coef: −0.02; 95% CI: −0.02, −0.01; *p* < 0.0001). Three other indicators of nutrition entered both models. A higher maternal weight-for-height category was associated with reduced odds of anemia (OR: 0.45; 95% CI: 0.25, 0.81; *p* = 0.007; second in dominance) and was also associated with higher hemoglobin, in a model run separately (*p* = 0.017; coef: 2.76; 95% CI: 0.50, 5.02; Supplementary Table S1A). Folic acid was positively associated with hemoglobin (*p* = 0.046; coef: 0.16; 95% CI: 0.003, 0.31; fourth in dominance) and entered the anemia model with *p* = 0.114. Vitamin A was associated with both lower odds of anemia (OR: 0.38; 95% CI: 0.15, 0.93; *p* = 0.035; fifth in dominance) and higher hemoglobin (coef: 3.39; 95% CI: 0.32, 6.46; *p* = 0.031; eighth in dominance). 

Additional variables emerged distinctively in the hemoglobin and anemia models. Hemoglobin was negatively associated with exposure to wood smoke (coef: −8.12; 95% CI: −12.4, −3.8; *p* < 0.0001; third in dominance) and positively associated with the lymphocyte count (coef: 3.18; 95% CI: 0.57, 5.79; *p* = 0.017; fifth in dominance), but no inflammation indicators were associated with anemia. Finally, higher parity was associated with increased odds of anemia (OR: 1.15; 95% CI: 1.01, 1.30; *p* = 0.030; third in dominance).

When exploring associations of Hb and anemia with other iron indicators, we found that Hb had positive associations with serum iron (coef: 0.33, 95% CI: 0.16, 0.49, *p* < 0.0001, second in dominance) and hepcidin (coef: 0.17, 95% CI: 0.02, 0.31; *p* = 0.023; sixth in dominance) and a negative association with sTfR (coef: −0.55, 95% CI: −0.92, −0.18; *p* = 0.004; fourth in dominance) (Supplementary Table S1B–D). Decreased odds of anemia were associated with higher serum iron (OR: 0.92, 95% CI: 0.87, 0.98, *p* = 0.006), but neither hepcidin (*p* = 0.097) nor sTfR (*p* = 0.171) were associated with anemia (Supplementary Table S2). 

Logistic regression models for infections (Table 7) revealed that the odds of caries were lowered with iron supplements (OR: 0.36; 95% CI: 0.15, 0.87; *p* = 0.22) and with higher serum iron (OR = 0.93; 95% CI: 0.87, 1.00; *p* = 0.036), but the odds increased with higher sTfR (OR: 1.11; 95% CI: 1.02, 1.22; *p* = 0.020). Taking iron supplements was also associated with lower odds of bacterial vaginosis (OR: 0.43; 95% CI: 0.19, 0.95; *p* = 0.037) but with increased odds of vaginal infection by the protozoa *Trichomonas* (OR: 2.36; 95% CI: 1.06, 5.26; *p* = 0.035). Higher hemoglobin was associated with increased odds of bacteriuria (OR: 1.04; 95% CI: 1.01, 1.08; *p* = 0.009), and higher serum iron with increased odds of *Ascaris* infection (OR = 1.06; 95% CI: 1.01, 1.12; *p* = 0.026). No associations were found between supplementation or iron status indicators with *Lactobacillus, Bacteroides/Gardnerella*, and *Mobiluncus*, or with scabies, vaginal diplococcus, intestinal hookworm, or *Trichuris*. 

#### 3.3.4. Distinctive Nature of Anemia and Iron Status Indicators

Based on a summary of the regression models shown in Table 4, Table 5 and Table 6, Table 8 shows that anemia and each iron status indicator was associated with a distinct set of nutritional, inflammatory, supplementation, and maternal factors. Hemoglobin and serum iron were associated with the largest range of factors, whereas sTfR was associated with only three factors. Table 8 summarizes the specific associations and their directions. 

## 4. Discussion

The comprehensive set of maternal nutritional status and inflammation biomarkers, infections, and extrinsic and intrinsic maternal factors, integrated in a conceptual framework, allowed us to better understand the complex interplay of these factors with anemia and iron status in our MINDI cohort and to provide insights into the lack of a maternal response to iron supplementation and association of iron supplementation with maternal infections. Hb was associated with the largest range of factors, demonstrating the multifactorial pathogenesis of anemia, and confirming the role of Hb as an indicator of overall maternal health [48]. Several key findings emerged:

- Anemia and each iron status indicator were associated with a distinct set of nutritional, inflammatory, and maternal factors and supplements;
- Wood smoke was an underreported extrinsic maternal factor that emerged as a risk factor for anemia;
- Plasma volume was an important intrinsic maternal factor, where greater plasma volume expansion contributed to the risk of anemia whereas low plasma volume likely led to the underestimation of the prevalence of anemia in this MINDI cohort;
- Inflammation indicators were not directly associated with decreasing Hb or increasing anemia but did contribute to the regulation of iron metabolism (evident in models for ferritin, serum iron, and hepcidin), with different inflammatory indicators (lymphocytes, monocytes, and CRP) associated with individual iron status parameters;
- Iron deficiency contributed to the risk of anemia as evidenced by the association of ferritin with hemoglobin and anemia;
- Undernutrition contributed to anemia, supported by associations of several nutritional indicators (low maternal weight-for-height, folic acid, and vitamin A) with Hb and anemia, by RBC indices, and by evidence of impaired erythropoiesis;
- Length of iron supplementation was not associated with anemia or with any other iron status indicator. However, the intake of higher amounts of an MNS supplement containing macro- and micronutrients was associated with higher ferritin and hepcidin concentrations;
- Iron supplements were associated with the presence of specific infections as women taking iron supplements had lower odds of caries and bacterial vaginosis but higher odds of vaginal trichomoniasis.

### 4.1. Neglected Maternal Factors Associated with Iron Status Indicators

We uncovered two overlooked maternal factors, plasma volume and wood smoke, that were associated with hemoglobin and anemia. Plasma volume expansion promotes blood flow to the uterus and nutrient delivery for normal fetal growth and development but is also critical when assessing anemia in pregnancy [81]. As a consequence, WHO hemoglobin cut-offs are lower in pregnant women to account for the physiological dilution due to the expected plasma volume expansion [1]. However, the lack of plasma volume expansion is rarely considered in pregnant women or in research on anemia during pregnancy [82,83]. In our study, even though anemic women had a higher plasma volume than non-anemic women, virtually all women in the second and third trimester had a low plasma volume that fell below the proposed cut-offs [54], suggesting that the prevalence of anemia would be even higher if plasma volume expansion had occurred in our MINDI cohort. We also suspect that underdiagnosis of anemia due to insufficient plasma volume expansion may be an overlooked issue in other studies and should be controlled for in future investigations.

In our study, exposure to wood smoke was negatively associated with Hb concentration. Previously, we had reported that the duration of exposure to wood smoke was positively associated with CRP in this MINDI cohort [44]. There is growing evidence that wildfire smoke induces inflammation in pregnant women, indicated by elevated CRP, IL8, IL6, MCP-1, neutrophils, and monocytes [84], and that biofuel exposure in pregnant women from India has been associated with anemia [85]. Given that interventions that reduce the exposure to biofuels for cooking have demonstrated favorable health effects [86], public health interventions addressing wood smoke exposure during pregnancy may be warranted.

### 4.2. Nutritional and Inflammation-Related Iron Deficiencies in the MINDI Context

In the setting of increased physiological iron needs related to pregnancy [87], poor diets contribute to the nutritional origin of iron deficiency. However, in our MINDI cohort, a puzzling observation was that iron supplements were not associated with any of our iron status indicators. Statistical analyses for ferritin and serum iron uncovered associations with other nutritional indicators, notably folic acid and vitamin B12, which captured an important proportion of their variability. Interactions between folic acid and iron metabolism have been described, including evidence that erythropoietin enhances both iron and folic acid absorption [88], that the carrier protein that mediates folic acid intestinal absorption (heme-carrier protein 1) may be a heme transporter [89], and that alterations in iron metabolism can affect folate-mediated one-carbon metabolism [90]. An interaction between iron and B12 metabolism has also been documented, as iron therapy in women with IDA corrected coexisting B12 deficiency [91]. It has also been proposed that in combined iron and B12 deficiencies, iron is not used by erythroblasts due to ineffective erythropoiesis related to B12 deficiency [92], but the exact mechanisms for iron–B12 interactions remain to be fully explained.

In addition to nutritional deficiencies, iron restriction due to inflammation was present in the MINDI cohort. CRP had a positive association with ferritin and hepcidin, and higher CRP and monocytes were associated with lower serum iron as first and third in dominance, respectively. These associations of iron indicators with inflammation were expected [93] and provided evidence for the presence of iron restriction due to inflammation in the MINDI cohort. 

Iron restriction due to inflammation is defined as the maldistribution of iron that occurs when excess hepcidin sequesters iron in macrophages, leading to hypoferremia that limits the availability of iron, especially for erythropoiesis, in the presence of adequate/increased iron stores [94]. In the MINDI cohort, it is likely that iron deficiency and iron restriction coexist; not only is there a high prevalence of total body iron depletion, but the presence of inflammation further prevents iron repletion. We suggest a relative excess of hepcidin in this population, as hepcidin should be blunted because of pregnancy [32] and because of iron deficiency [95]. Currently, it is understood that, under normal conditions, hepcidin is suppressed during the second and third trimesters of pregnancy [96], with reported values up to 10-fold lower in the second trimester over a similar range of ferritin during normal pregnancy [33]. We did not observe this degree of suppression in our cohort, where the hepcidin/ferritin ratio was similar, with a median of 0.6 in the first and second trimesters, and slightly increased (instead of decreasing) to 0.8 in the third trimester, suggesting that hepcidin was not proportionally suppressed compared to ferritin despite the high prevalence of anemia and iron deficiency in the MINDI cohort. In fact, studies using the same hepcidin laboratory kit that we employed have described that hepcidin > 20 µg/L (present in 13.6% of our cohort) had a positive predictive value of 81.6% for the detection of non-response to iron therapy (Hb increase ≤1 g/dL) [97], and hepcidin < 10 µg/L in blood donors (present in 61.5% of our cohort) correctly identified ferritin < 15 µg/L in 85% of cases [98]. Therefore, our findings might suggest that lower hepcidin concentrations could indicate iron deficiency. On the other hand, elevated hepcidin was also present in this population and may be playing a role in the lack of responsiveness to iron supplementation. 

### 4.3. Multifactorial Origin of Anemia in the MINDI Cohort

Our findings showed that anemia in this cohort was predominantly associated with iron deficiency, as evidenced by dominance analyses. Ferritin was the main determinant of anemia, with higher concentrations decreasing its odds, and where decreased odds of anemia were associated with higher serum iron. Using a recently proposed cut-off of ferritin < 30 mg/L indicating severe iron deficiency in pregnancy [95,99], coexistent low ferritin and anemia were found in 35.2% of pregnant women (92.6% of anemic women). Our findings fall in the range of current estimated IDA in pregnancy in developing settings of 35–75% [99]. However, iron deficiency did not explain all our findings. RBC indices showed evidence of more hypochromia in anemic women but did not show the typical expected microcytosis or larger RDW observed in IDA [100] compared with non-anemic counterparts. Instead, MCV showed values with a trend towards macrocytosis, and other micronutrients such as vitamin A and folic acid entered models for anemia and/or Hb. 

The associations of low weight-for-height category and lower folate and vitamin A with both anemia and lower Hb suggest the presence of anemia of undernutrition, mimicking previously reported results in lactating women from the same cohort, where anemia of undernutrition was also evident [101]. The term ‘anemia of undernutrition’ is used to capture the range of nutritional deficiencies that contribute to anemia, and it encompasses iron and other macro- and micronutrient deficiencies [102]. The morphology of RBC in anemia of undernutrition has been described as normocytic, normochromic, or mildly hypochromic, and as presenting anisocytosis, but these features can be further modified by coexistent micronutrient deficiencies and infections [103]. A normal MCV in anemia of undernutrition may relate to the microcytic influence of iron deficiency counterbalancing the macrocytic effect of B12 and folate deficiency [104]. This description fits our MINDI population, where anemia was mainly normocytic, hypochromic, and with anisocytosis, and coexisted with multiple nutrient deficiencies and infections.

The pathophysiology of anemia of undernutrition has been associated with protein, iron, and vitamin deficiencies resulting in erythropoietin deficiency, bone marrow hypoplasia, and metabolic changes in the red cells, including decreased production and alteration in the red cell membrane with increased fragility [103,105]. Moreover, folic acid and vitamin B12 deficiencies impair DNA synthesis, producing ineffective erythropoiesis [106,107]. Also, protein malnutrition is known to considerably reduce sTfR in bone marrow and spleen cell populations in experimental malnutrition [108], and to produce bone marrow hypoplasia with a decrease in hematopoietic cells in experimental and human studies [105]. The MINDI cohort encompasses these characteristics, where vitamin B12/protein deficiencies may have impaired the erythropoietic activity, commonly associated with lower concentrations of sTfR [109], which we observed in the second trimester. 

Therefore, in addition to the lack of availability of iron for erythropoiesis, we believe that erythropoiesis was blunted in our cohort. We used sTfR as an indicator of erythropoiesis, as it is known to strongly correlate with erythropoietin during pregnancy [110], and to be low in bone marrow hypoproliferation [111]. Whereas associations of lower sTfR with higher Hb [112] and with the intake of iron supplementation in iron-deficient pregnant women have been demonstrated [19], we found elevated sTfR showing tissue iron deficiency only in 16.4%, contrasting with >40% prevalence of iron deficiency indicated by ferritin and serum iron. A possible explanation for this could be a physiological suppression of sTfR found by others during early pregnancy [63,110], which was observed in our cohort where sTfR was higher in the third trimester, and where the trimester positively predicted sTfR as first in dominance. However, Beguin et al. found that, despite its suppression during pregnancy, sTfR was higher in pregnant women with low iron stores compared with those with marginal or normal ferritin concentrations [110]. Therefore, our findings would support a lack of an erythropoietic response.

Another important micronutrient for red cell formation is vitamin D, known to support this process by decreasing pro-inflammatory cytokines and by increasing erythroid progenitor cells [113]. We expected to find a positive association of vitamin D with sTfR; instead, vitamin D emerged with negative associations with sTfR and with serum iron. Similarly, Thomas et al. found lower vitamin D concentrations with higher erythropoietin and elevated sTfR in pregnant adolescents [114]. Others found in vitamin D-deficient adolescents that vitamin D supplementation reduced serum iron [115], and in multiple studies, vitamin D was inconsistently shown to reduce hepcidin [116]. These associations indicate that further study is needed to clarify the mechanisms.

Supporting the lack of protein for erythropoietic process, we have previously reported that, although the total WBC counts were similar among women with normal and low protein status measured using retinol-binding protein (RBP), protein-deficient mothers had lower eosinophil and basophil counts, as well as lower IL-4 [43]. In the current analyses, we found an unexpected positive association of lymphocyte counts with Hb. A decreased immune response expressed as lower lymphocytes in women with lower Hb supports the malnutrition origin of anemia in our population, given that lymphocyte number and response to infections have been shown to be decreased during experimental malnutrition [117,118], and in adults with B12 deficiency [119]. Impaired immunity, and specifically lower lymphocyte counts, have been reported for protein-malnourished individuals [105], and experimental studies have shown lower lymphocyte counts during iron [120] and vitamin A [121] deficiencies, both of which emerged as negative predictors for hemoglobin in our study.

The positive association of lymphocyte counts with Hb was counter to our hypothesis of decreased Hb with inflammation. Anemia of inflammation was suspected, given that all women had at least two mild–moderate chronic infections [45], and given the lack of association of anemia with iron supplementation. Anemia of inflammation has been described as usually mild, presenting with erythrocytes of normal size (normocytic) and Hb content (normochromic), decreased serum iron but replete iron stores (ferritin > 100 mg/L), elevation of inflammatory markers (CRP, IL-6), and elevated hepcidin [94,122]. Despite the presence of iron restriction due to inflammation, the impact of inflammation on Hb and anemia was less evident. We had previously reported that in this population, inflammation as measured using CRP was positively associated with certain infections (caries, hookworm, vaginal diplococcal infection), but negatively associated with others (*Ascaris*, vaginal *Lactobacillus*, and *Bacteroides/Gardnerella*) [44]. We propose that in the presence of multiple infections associated with Th2 cytokine profiles, CRP may not be the best indicator of inflammation when studying anemia, as shown in the lack of association of CRP with anemia or Hb. Moreover, given that indicators of a pro-inflammatory response did not differ between anemic and non-anemic women, that the association of hepcidin and lymphocytes with Hb was positive, and that neither hepcidin nor CRP were associated with anemia, these findings would not support inflammation as a direct predictor of anemia in the MINDI cohort.

### 4.4. Impacts of Iron and MNS Supplementation on Anemia, Iron Status Indicators, and Infections

Despite the known benefit of iron supplementation in the reduction of maternal anemia when compared with no treatment in low–medium income countries [123], we showed that the duration of iron supplementation was not associated with Hb, ferritin, sTfR, serum iron or hepcidin. In contrast, a higher intake of MNS/d (observed in undernourished women) positively predicted ferritin and hepcidin but did not enter models for other iron status indicators. Consistent with this, epidemiological evidence has shown that iron supplementation with additional micronutrient supplementation leads to a greater effect on ferritin concentrations, compared with iron alone [122]. Our findings suggest that for the improvement of iron status in the MINDI cohort, MNS would have a greater impact than iron supplementation alone. Others have shown that iron supplementation alone increases pro-inflammatory cytokines, in particular IL-6, and decreases ferroportin production and contributes to iron overload and an increased risk of infection. [124].

Additionally, we found that iron supplementation differentially altered the odds of specific infections. In our cohort, iron supplementation was associated with lower odds of caries in agreement with the association of IDA during pregnancy as a risk factor for the incidence of dental caries in pregnant women from Brazil [125]. Iron supplements also decreased the odds of bacterial vaginosis but increased the odds of vaginal trichomoniasis, reflecting the important role of iron in the maintenance of balanced vaginal microflora [126]. We found that a higher iron status, indicated by higher serum iron, increased the odds of ascariasis, given that iron deficiency is both a risk factor for soil-transmitted helminths, in addition to being a consequence of some intestinal parasitic infections [127]. The odds of bacteriuria were also increased with higher hemoglobin, in line with the most common causal agent of urinary tract infection, *Escherichia coli*, having at least ten iron uptake systems [128]. The complex interaction between iron homeostasis and infections, where competition for iron happens between the host and infectious organisms, has been extensively reviewed, stressing the need to treat infections before iron administration in endemic populations [129] like ours.

## 5. Strengths and Limitations

This is the first study to explore a broad set of etiological factors that have been associated with anemia during pregnancy in mothers experiencing multiple infections, nutrient deficiencies, and inflammation. We also controlled for usually neglected factors such as plasma volume and the exposure to wood smoke, as well as common maternal gastrointestinal and vaginal infections with implications for public health. From the statistical point of view, although the use of GLM regressions for iron status indicators, and dominance analyses are not common in anemia-related research, they were able to capture the rank order of variables and contributed to the interpretation of our findings under MINDI conditions.

We also acknowledge several limitations. This was a cross-sectional study that precludes inferring causation. The emergence of low plasma volume during pregnancy in our MINDI cohort likely led to an underestimation of anemia and may have equally impacted other iron status indicators. The intake of supplements was subject to recall bias and may have been shared with other family members. 

## 6. Conclusions

This study highlights the complexity of interactions among MINDI factors in a vulnerable and malnourished population and highlights the challenges of interpreting iron status indicators in marginalized pregnant women in low- and middle-income countries. It is clear from our findings that anemia in the Ngäbe-Buglé cohort of pregnant women was multifactorial, with anemia of undernutrition playing an important role. Athough inflammation could be playing a role in the development of anemia, we did not find evidence of a direct association, but an indirect link through iron restriction due to inflammation, where inflammation was characterized by multiple biomarkers and where no single biomarker was sufficient to describe associations with individual iron status indicators.

Given the lack of association of iron supplementation with Hb or ferritin, the approach for preventing and treating anemia and iron deficiency in indigenous women with MINDI needs to be re-evaluated. Caution is needed in the use of iron supplements during pregnancy within a MINDI context, as iron supplements were associated with increased odds of certain infections. In contrast, associations of iron indicators with weight-for-height, vitamin A, folic acid, and B12 highlight the need for a more holistic nutritional approach. A positive association of MNS with ferritin suggests that this type of supplementation could be more helpful, but compliance with appropriate dosages needs to be encouraged.

## Figures and Tables

**Table 1:** Iron status indicators and plasma volume by trimester.

	1st Trimester (*n* = 26)	2nd Trimester (*n* = 80)	3rd Trimester (*n* = 107)	
Iron Status Indicators	Median (IQR) or %	Median (IQR) or %	Median (IQR) or %	*p* ^3^
Hemoglobin, g/L	118.5 (108.0, 126.0) ^a^	111.0 (104.0, 115.5) ^b^	113.0 (106.0, 141.0) ^b^	0.003
Hemoglobin < 110 g/L	30.8%	43.7%	35.5%	0.372
Hematocrit, %	35.9 (33.3, 38.6) ^a^	34.5 (32.4, 36.0) ^b^	35.4 (33.5, 37.6) ^ab^	0.014
<33%	15.4%	28.7%	18.7%	0.177
Ferritin, µg/L	31.8 (18.2, 50.6) ^a^	16.1 (7.5, 26.1) ^b^	8.9 (5.1, 17.5) ^c^	0.0001
<30 µg/L	46.1%	82.5%	91.6%	<0.0001
<15 µg/L	7.7%	45.0%	70.1%	<0.0001
Serum iron, µmol/L	11.7 (8.1, 14.2)	8.7 (6.0, 13.4)	8.1 (5.4, 13.6)	0.078
<8.9 µmol/L	34.6%	52.5%	56.1%	0.145
sTfR, mg/L	4.0 (3.2, 5.4) ^b^	4.4 (3.0, 6.2) ^b^	5.0 (4.5, 7.8) ^a^	0.0001
>8.3 mg/L	7.7%	11.2%	22.4%	0.060
<3.0 mg/L	11.5%	22.5%	4.7%	0.001
Hepcidin, µg/L	15.1 (10.1, 24.4) ^a^	8.7 (5.7, 13.4) ^b^	7.2 (5.0, 10.5) ^b^	0.0001
Hepcidin/ferritin ratio	0.6 (0.4, 0.8) ^b^	0.6 (0.3, 1.2) ^b^	0.8 (0.5, 1.2) ^a^	0.016
	**Mean ± SD or %**	**Mean ± SD or %**	**Mean ± SD or %**	***p* ^1^**
Plasma volume, L	1.97 ± 0.17 ^b^	2.10 ± 0.18 ^a^	2.17 ± 0.20 ^a^	<0.0001
Low plasma volume ^2^	61.5%	98.7%	100%	<0.0001

^1^ One-way ANOVA was used to assess differences in plasma volume by trimester. ^2^ Low plasma volume: <2 L in the 1st, <2.6 L in the 2nd, and <2.8 L in the 3rd trimester. ^3^ The Kruskal–Wallis test was used for continuous variables. For dichotomous variables, the Chi^2^ test was used if all cells in 3 × 2 tables had expected values > 5, or Fisher’s exact test if any cell contained values ≤ 5. Superscripts ^a, b, c^ indicate a higher value (a > b > c) when *p* < 0.05, using one-way ANOVA on normalized log-transformed variables. WHO cut-offs for anemia [57], low hematocrit [58], and low serum iron [79] are reported. For sTfR, the manufacturer RAMCO’s cut-off was used. As no WHO cut-off for low ferritin during the second and third trimester is established, the cut-off for non-pregnant women [15]), and the value used by the Panamanian Ministry of Health at the time of the study [62], are reported.

**Table 2:** Comparisons of maternal characteristics and nutritional status indicators between mothers without and with anemia.

		Non-Anemic (Hb ≥ 110 g/L)	Anemic (Hb < 110 g/L)	
General Characteristics ^1^	Freq. ^2^	Median (IQR) or %	Median (IQR) or %	*p* ^3^
n		62.0%	38.0%	
Age, years		23 (18–30)	25 (21–30)	0.107
Calculated plasma volume, L		2.06 (1.96, 2.20)	2.14 (1.86, 2.29)	0.001
Low plasma volume		96.2%	29.6%	0.199
Parity		2 (1, 5)	4 (2, 6)	0.007
Wood smoke exposure	195	88.6%	96.3%	0.051
Taking iron	163	75.8%	77.8%	0.736
Months taking iron		2 (0.5, 3.5)	2 (1, 3)	0.840
^4^ Taking MNS	108	48.5%	54.3%	0.408
MNS, tbsp/d		0 (0, 2)	1 (0, 4)	0.282
**Nutritional indicators**				
^4^ Weight-for-height category				
Underweight	21	8.3%	12.3%	0.014
Normal weight	143	62.1%	75.3%	
Overweight/obese	49	29.5%	12.3%	
^5^ RBP, mg/L		49.0 (28.3, 82.9)	46.5 (29.5, 89.4)	0.668
<30 mg/L	57	27.5%	25.9%	0.804
IGF-1, µg/L		19.3 (8.5, 46.6)	20.1 (9.2, 48.1)	0.714
^6^ Low IGF-1	160	76.7%	75.3%	0.812
**Micronutrient deficiencies**				
Folic acid <10 µmol/L	51	18.2%	33.3%	0.012
B12 < 150 pmol/L	181	82.6%	88.9%	0.211
Vit. D < 50 nmol/L	138	62.1%	69.1%	0.298
^7^ Vit. A < 1.05 µmol/L	87	36.9%	48.7%	0.091
**Iron indicators**				
Hemoglobin (g/L)		117 (113, 122)	104 (99, 106)	0.0001
Ferritin, µg/L^2^		17.4 (8.7, 29.4)	7.9 (3.3, 16.6)	0.0001
<30 µg/L	176	76.5%	92.6%	0.003
<15 µg/L	113	43.2%	69.1%	<0.0001
Serum iron, µmol/L		10.3 (7.1, 16.0)	6.5 (4.4, 9.3)	0.0001
<8.9 µmol/L	111	40.9%	70.4%	<0.0001
sTfR, mg/L		5.0 (3.8, 6.4)	5.7 (3.3, 8.1)	0.270
>8.3 mg/L	35	12.1%	23.5%	0.030
<3.0 mg/L	11	11.4%	13.6%	0.391
Hepcidin, µg/L^2^		8.4 (5.5, 15.0)	8.0 (6.2, 11.6)	0.539
Hepcidin/ferritin ratio		0.6 (0.3, 0.9)	1.1 (0.6, 1.9)	0.0001
**Other RBC indices**				
Hematocrit, %		36.3 (35.2, 37.8)	32.6 (31.2, 33.9)	0.0001
<33%	47	0.8%	56.8%	<0.0001
MCV, fL		94.6 (91.3, 97.5)	93.3 (86.6, 96.8)	0.013
Macrocytosis (MCV > 100 fL)	25	12.9%	9.9%	0.509
Normocytosis	180	87.1%	80.2%	0.178
Microcytosis (MCV < 80 fL)	8	0%	9.9%	<0.0001
MCHC, g/L		323 (316, 330)	314 (308, 324)	0.0001
Hypochromia (MCHC < 320 g/L)	102	37.1%	65.4%	<0.0001
RDW-SD, fL (*n* = 198)		45.9 (43.6, 46.9)	45.9 (43.6, 48.0)	0.705
Anisocytosis (>46 fL)	69	32.6%	39.1%	0.361

IQR: interquartile range, MNS: multiple nutrient supplement; MCV: mean corpuscular volume. ^1^ Total observations *n* = 213, unless otherwise specified; ^2^ frequencies of categorical variables are presented; ^3^ the Chi^2^ test was used if all cells in 3 × 2 or 2 × 2 tables had expected values > 5, or Fisher’s exact test if any cell contained values ≤ 5. Comparisons were made between the proportion of positive cases in non-anemic vs. anemic pregnant women. Differences in the distribution of continuous variables were calculated using the Kruskal–Wallis test. ^4^ Weight-for-height for gestational age were defined using PAHO standards [80]. ^5^ RBP: serum samples, *n* = 212; non-anemic: *n* = 131, 61.8%; anemic: *n* = 81, 38.2%. ^6^ IGF-1 < 49.6 µg/L in the 1st, <41.1 in the 2nd, and <61.7 in the 3rd trimester [69]. ^7^ Vitamin A: serum samples, *n* = 210; non-anemic: *n* = 130, 61.9%; anemic: *n* = 80, 38.1%.

**Table 3:** Comparisons of maternal infections and biomarkers of inflammation between mothers without and with anemia.

		Non-Anemic (Hb ≥ 110 g/L)	Anemic (Hb < 110 g/L)	
Infections ^1^	Freq. ^2^	% or Median (IQR)	% or Median (IQR)	*p* ^3^
Caries	42	17.4%	23.5%	0.283
Scabies	37	18.9%	14.8%	0.441
^4^ Bacteriuria	54	75.9%	24.1%	0.012
^5^ *Lactobacillus*	113	52.3%	55.7%	0.629
^5^ *Bacteroides/Gardnerella*	198	95.4%	91.1%	0.207
^5^ *Mobiluncus*	174	78.0%	89.9%	0.029
^5^ *Trichomonas*	52	71.2%	82.3%	0.071
Vaginal yeast	53	23.5%	27.2%	0.547
^5^ *Diplococcus*	43	17.4%	25.3%	0.168
^6^ *Ascaris*	39	30.0%	37.5%	0.408
^6^ Hookworm	68	58.7%	52.5%	0.515
^6^ *Trichuris*	15	13.7%	10.0%	0.394
**Inflammation biomarkers**				
CRP, mg/L (*n* = 213)		3.3 (1.3, 6.7)	4.1 (1.6, 7.4)	0.354
>5 mg/L	76	33.3%	39.5%	0.361
White blood cells (WBCs) × 10^3^/mm^3^				
Total WBCs		8.61 (7.45, 10.30)	8.08 (6.97, 9.13)	0.030
Neutrophils		5.87 (4.67, 7.06)	5.21 (4.29, 6.29)	0.023
Lymphocytes		1.99 (1.70, 2.37)	1.91 (1.70, 2.18)	0.231
Monocytes		0.37 (0.31, 0.45)	0.34 (0.29, 0.41)	0.138
Eosinophils		0.37 (0.18, 0.53)	0.32 (0.19, 0.45)	0.548
Basophils		0.03 (0.02, 0.04)	0.03 (0.02, 0.04)	0.898
^7^ Cytokines, pg/mL (*n* = 212)				
IL-10		1.6 (0.1, 6.7)	1.0 (0.1, 3.8)	0.027
IL-13		1.6 (0.2, 9.1)	1.6 (0.1, 6.6)	0.097
IFN-γ		5.5 (1.2, 15.3)	2.5 (0.8, 13.6)	0.132

CRP: C-reactive protein; IQR: interquartile range. ^1^ Total observations *n* = 213, unless otherwise specified. ^2^ Frequencies of categorical variables are presented. ^3^ The Chi^2^ test was used if all cells in 3 × 2 or 2 × 2 tables had expected values > 5, or Fisher’s exact test if any cell contained values ≤ 5. Comparisons were made between the proportion of positive cases in non-anemic vs. anemic pregnant women. Differences in the distribution of continuous variables were calculated using the Kruskal–Wallis test. ^4^ Urine samples, *n* = 208; non-anemic: *n* = 128, 61.5%; anemic: *n* = 80, 38.5%. ^5^ Vaginal samples, *n* = 211; non-anemic: *n* = 132, 62.6%; anemic: *n* = 79, 37.4%g. ^6^ Fecal samples, *n* = 120; non-anemic: *n* = 80, 66.7%; anemic: *n* = 40, 33.3%. ^7^ Other cytokines (IL-1β, IL-4, IL-6, IL-12, IL-17, and TNF-α) did not differ between anemic and non-anemic women (*p* > 0.15).

**Table 4:** GLM regression models for ferritin and hepcidin in pregnant women and MINDI variables.

**(A)**					
Ferritin, ug/L *	**Coef.**	***p***	**95% CI**	**Standardized ** **Domin. Stat**	**Ranking**
Trimester	−9.34	<0.0001	−13.37, −5.31	0.53	1
B12, pmol/L *	0.06	0.046	0.001, 0.11	0.20	2
Folic acid, nmol/L	0.27	0.088	−0.04, 0.58	0.12	3
Months taking iron	0.55	0.168	−0.23, 1.32	0.06	4
CRP, mg/L	0.38	0.043	0.01, 0.75	0.05	5
MNS, tbsp/d	1.60	0.016	0.30, 2.91	0.03	6
^1^ Weight-for-height category	2.31	0.111	−0.53, 5.16	0.01	7
Constant	20.09	0.006	5.72, 34.45		
**(B)**					
Hepcidin *	**Coef.**	***p***	**95% CI**	**Standardized ** **Domin. Stat**	**Ranking**
Trimester	−4.18	<0.0001	−5.94, −2.42	0.43	1
B12, pmol/L *	0.02	0.062	−0.001, 0.05	0.17	2
CRP, mg/L	0.31	0.001	0.12, 0.49	0.17	3
Folic acid, nmol/L	0.14	0.034	0.01, 0.26	0.12	4
MNS, tbsp/d	0.87	0.013	0.19, 1.55	0.04	5
Parity	0.30	0.111	−0.07, 0.67	0.04	6
Months taking iron	0.41	0.054	−0.01, 0.82	0.04	7
Constant	11.66	<0.0001	5.32, 17.99		

^1^ Weight-for-height for gestational age category: 0: underweight, 1: normal, 2: overweight/obese for gestational age. (**A**) Model for ferritin, µg/L: *n* = 213, overall fit statistic = 0.246, VIF = 1.24. (**B**) Model for hepcidin, µg/L: *n* = 213, overall fit statistic = 0.221, VIF = 1.23. * Variable has been winsorized.

**Table 5:** GLM regression models for serum iron and sTfR in pregnant women with MINDI.

**(A)**					
**Serum Iron, umol/L**	**Coef.**	***p***	**95% Conf.**	**Standardized** **Domin. Stat**	**Ranking**
Monocytes ×10^3^/mm^3^ *	−14.49	<0.0001	−19.10, −9.87	0.29	1
Folic acid, nmol/L	0.19	0.004	0.06, 0.31	0.28	2
CRP, mg/L	−0.30	<0.0001	−0.40, −0.20	0.25	3
Vitamin B12, pmol/L *	0.03	0.016	0.005, 0.05	0.09	4
Trimester	−0.61	0.360	−1.91, 0.69	0.04	5
Vitamin D, nmol/L	−0.06	0.046	−0.11, −0.001	0.03	6
Months taking iron	0.15	0.489	−0.28, 0.59	0.01	7
Constant	15.68	<0.0001	10.44, 20.93		
**(B)**					
**sTfR, mg/L**	**Coef.**	***p***	**95% CI**	**Standardized ** **Domin. Stat.**	**Ranking**
Trimester	1.04	<0.0001	0.54, 1.55	0.38	1
Vitamin D, nmol/L	−0.03	0.022	−0.05, −0.004	0.15	2
^1^ Weight-for-height category	−0.79	0.030	−1.51, −0.07	0.12	3
CRP, mg/L	0.08	0.085	−0.01, 0.18	0.11	4
Eosinophils ×10^3^/mm^3^ *	−1.30	0.067	−2.68, 0.09	0.10	5
Parity	−0.14	0.052	−0.29, 0.001	0.08	6
Animal-source foods, portions/wk	0.09	0.173	−0.04, 0.23	0.05	7
Constant	6.62	<0.0001	4.28		

^1^ Weight-for-height for gestational age category: 0: underweight, 1: normal, 2: overweight/obese. (**A**) Model for serum iron (µmol/L): *n* = 213, overall fit statistic = 0.136, VIF = 1.23. (**B**) Model for sTfR (mg/L): *n* = 212, overall fit statistic = 0.117, VIF = 1.04. * Variable has been winsorized.

**Table 6:** Logistic regression model for anemia and linear regression model for Hb in pregnant women with MINDI.

**(A)**					
**Anemia (Hb <110 g/L)**	**OR**	***p***	**95% CI**	**Standardized** **Domin. Stat.**	**Ranking**
Ferritin, µg/L *	0.96	0.001	0.93, 0.98	0.32	1
^1^ Weight-for-height category	0.45	0.007	0.25, 0.81	0.16	2
Parity	1.15	0.030	1.01, 1.30	0.11	3
Folic acid, nmol/L	0.96	0.114	0.92, 1.01	0.11	4
Vitamin A, µmoll/L	0.38	0.035	0.15, 0.93	0.11	5
^2^ Low plasma volume	0.13	0.033	0.02, 0.84	0.08	6
^3^ Wood smoke exposure	3.31	0.129	0.70, 15.50	0.07	7
Trimester	0.73	0.272	0.42, 1.28	0.03	8
Constant	113.18	0.002	5.82, 2200.15		
**(B)**					
Hemoglobin (g/L) *	**Coef.**	***p***	**95% CI**	**Standardized** **Domin. Stat.**	**Ranking**
Plasma volume, mL	−0.02	<0.0001	−0.02, −0.01	0.30	1
Ferritin, µg/L *	0.20	<0.0001	0.12, 0.27	0.27	2
^3^ Wood smoke exposure	−8.12	<0.0001	−12.43, −3.82	0.14	3
Folic acid, nmol/L	0.16	0.046	0.003, 0.31	0.09	4
Lymphocytes × 10^3^/mm^3^	3.18	0.017	0.57, 5.79	0.06	5
Parity	−0.36	0.126	−0.83, 0.10	0.06	6
Trimester	3.28	0.001	1.34, 5.22	0.04	7
Vitamin A, µmol/L	3.39	0.031	0.32, 6.46	0.04	8
Constant	134.76	<0.0001	118.39, 151.12		

(A) Model *n* = 210, overall fit statistic = 0.165, VIF = 1.17, AUC = 0.773. (B) Model *n* = 209, adj. R^2^ = 0.33, overall fit statistics = 0.36, VIF = 1.13. ^1^ Weight-for-height for gestational age category: 0: underweight, 1: normal, 2: overweight/obese. ^2^ Low plasma volume: <2 L in the 1st, <2.6 L in the 2nd, and <2.8 L in the 3rd trimester. ^3^ Wood smoke exposure categorized as 0: no exposure, 1: exposure. * Variable has been winsorized. Note: We used the binary variable “low plasma volume” in the logistic model for anemia, for allowing the inclusion of the weight-for-height category (collinear with plasma volume as continuous variable). For the linear hemoglobin model, the continuous variable for plasma volume was used. A separate model including weight-for-height while excluding plasma volume was run (Table S1A).

**Table 7:** Logistic regression models for the presence of infections with iron status indicators (tested separately), adjusting for trimester.

Presence of Infections ^1^	*n*	Iron Intake (Yes/No) or Iron Status Indicator (Continuous)	OR	95% CI	*p*
^2^ Caries	213	Taking iron supplements	0.36	0.15, 0.87	0.022
^2^ Caries		Serum iron	0.93	0.87, 1.00	0.036
^2^ Caries		sTfR	1.11	1.02, 1.22	0.020
^3^ Bacterial vaginosis	211	Taking iron supplements	0.43	0.19, 0.95	0.037
Vaginal trichomoniasis	211	Taking iron supplements	2.36	1.06, 5.26	0.035
Bacteriuria	208	Hemoglobin	1.04	1.01, 1.08	0.009
*Ascaris*	120	Serum iron	1.06	1.01, 1.12	0.026

^1^ Presence of infections were coded as 0: absent, 1: present. Logistic regression models for other microorganisms were run, resulting in associations with supplementation/iron indicators with *p*-values > 0.05: *Lactobacillus, Bacteroides/Gardnerella*, and *Mobiluncus* (tested separately), and scabies, vaginal diplococcus, intestinal hookworm, and *Trichuris.* ^2^ Models for caries were run individually with different iron status indicators. ^3^ Bacterial vaginosis was calculated as per Nugent score = Bacteroides/Gardnerella score + (4 − Lactobacillus score) + (Mobiluncus score/2) [55].

**Table 8:** Synopsis of associations of nutrients, inflammation indicators, supplements, and maternal factors that entered models for anemia and iron status indicators with *p* < 0.05.

Iron Status Indicator	Nutritional Indicators	Inflammation	Supplements	Maternal Factors	Predictors (*p* < 0.05)
OR of Anemia	Ferritin (reduced) Serum iron (reduced) Vitamin A (reduced)	None	None	Weight-for-height (reduced) Plasma volume (increased) Parity (increased)	6
Hemoglobin	Ferritin (+) Serum iron (+) Hepcidin (+) Vitamin A (+) Folic acid (+)	Lymphocytes (+)	None	Trimester (+) Plasma volume (−) Wood smoke (−) Weight-for-height category (+)	10
Ferritin	Vitamin B12 (+)	CRP (+)	MNS (+)	Trimester (−)	4
Hepcidin	Folic acid (+)	CRP (+)	MNS (+)	Trimester (−)	4
Serum iron	Folic acid (+) Vitamin B12 (+) Vitamin D (−)	CRP (−) Monocytes (−)	None	None	5
sTfR	Vitamin D (−)	None	None	Trimester (−) Weight-for-height category (−)	3

(+) Indicates positive associations, and (−) indicates negative associations with the nutritional status indicator in column 1. B12, vitamin B12; CRP, C-reactive protein; MNS, multiple nutrient supplement; OR, odds ratio; sTfR, serum transferrin receptor.

## Data Availability

The data are not publicly available because participants did not sign an informed consent form stating that data would be publicly available; neither was this possibility discussed with the indigenous communities or the Ethical Board in Panama.

## Supplementary Materials

The following supporting information can be downloaded at: https://www.mdpi.com/article/10.3390/nu16111748/s1, Table S1: Additional linear regression models for Hemoglobin, Table S2: Additional logistic regression models for Anemia.
