# Supplementary material for: Multiple Infections, Nutrient Deficiencies, and Inflammation as Determinants of Anemia and Iron Status during Pregnancy: The MINDI Cohort
Source: Nutrients. 2024 Jun 2;16(11):1748. doi: 10.3390/nu16111748 (PMC11174717; doi:10.3390/nu16111748)
Supplement: Supplementary file 1 [file nutrients-16-01748-s001.zip › nutrients-3008631-supplementary.pdf]

Supplementary material

Table S1. Additional linear regression models for Hemoglobin: (A) Including weight-for-height category instead of plasma volume. (B) Including serum iron instead of ferritin. (C) Including hepcidin instead of ferritin. (D) Including sTfR instead of ferritin.

(A) Hemoglobin (g/L) *	Coef.	*p*	95% CI	Standardized Domin. Stat.	Ranking
Ferritin, µg/L *	0.19	<0.0001	0.11, 0.27	0.37	1
[1] Wood smoke exposure	-6.05	0.010	-10.62, -1.48	0.15	2
Folic acid, nmol/L	0.20	0.017	0.04, 0.36	0.13	3
[2] Weight-for-height category	2.76	0.017	0.50, 5.02	0.11	4
Parity	-0.55	0.030	-1.05, -0.05	0.10	5
Lymphocytes $\times 10^3$/mm^3	2.68	0.062	-0.14, 5.49	0.07	6
Vitamin A, µmol/L	3.31	0.047	0.04, 6.57	0.05	7
Trimester	1.52	0.136	-0.48, 3.52	0.02	8
Constant	93.64	<0.0001	82.06, 105.21		
**(B) Hemoglobin (g/L) ***	**Coef.**	***p***	**95% CI**	**Standardized Domin. Stat.**	**Ranking**
[3] Plasma volume, mL	-0.02	<0.0001	-0.02, -0.01	0.34	1
Serum iron, µmol/L	0.33	<0.0001	0.16, 0.49	0.22	2
[1] Wood smoke exposure	-7.77	0.001	-12.20, -3.34	0.15	3
Folic acid, nmol/L	0.16	0.055	-0.003, 0.32	0.10	4
Parity	-0.44	0.073	-0.92, 0.04	0.07	5
Lymphocytes $\times 10^3$/mm^3	2.91	0.033	0.23, 5.59	0.06	6
Vitamin A, µmol/L	3.46	0.031	0.31, 6.62	0.05	7
Trimester	1.28	0.178	-0.59, 3.14	0.01	8
Constant	139.57	<0.0001	122.98, 156.17		
**(C) Hemoglobin (g/L) ***	**Coef.**	***p***	**95% CI**	**Standardized Domin. Stat.**	**Ranking**
[3] Plasma volume, mL	-0.02	0.000	-0.02, -0.01	0.37	1
[1] Wood smoke exposure	-8.58	0.000	-13.10, -4.06	0.18	2
Folic acid, nmol/L	0.21	0.012	0.05, 0.37	0.13	3
Parity	-0.52	0.040	-1.01, -0.02	0.09	4
Lymphocytes $\times 10^3$/mm^3	3.19	0.023	0.45, 5.93	0.08	5
Hepcidin, µg/L	0.17	0.023	0.02, 0.31	0.08	6
Vitamin A, µmol/L	3.57	0.030	0.35. 6.79	0.05	7
Trimester	2.13	0.036	0.15, 4.12	0.02	8
Constant	138.36	0.000	121.07, 155.66		
**(D) Hemoglobin (g/L) ***	**Coef.**	***p***	**95% CI**	**Standardized Domin. Stat.**	**Ranking**
[3] Plasma volume, mL	-0.02	0.000	-0.03, -0.01	0.39	1
[1] Wood smoke exposure	-8.10	0.000	-12.59, -3.61	0.17	2
Folic acid, nmol/L	0.20	0.017	0.04, 0.35	0.12	3
sTfR, mg/L	-0.55	0.004	-0.92, -0.18	0.10	4
Parity	-0.52	0.036	-1.01, -0.03	0.09	5
Lymphocytes $\times 10^3$/mm^3	3.14	0.024	0.42, 5.86	0.07	6
Vitamin A, µmol/L	3.15	0.054	-0.06, 6.36	0.04	7
Trimester	2.06	0.037	0.13, 3.99	0.02	8
Constant	147.24	0.000	130.22, 164.26		

(A) Model n = 210, overall fit statistic = 0.275, VIF = 1.11. (B) Model n = 210, overall fit statistics = 0.321, VIF = 1.09. (C) Model n = 210, overall fit statistics = 0.29, VIF = 1.11. (D) Model n = 210, overall fit statistics = 0.30, VIF = 1.10. [1] Wood smoke exposure categorized as 0: no exposure, 1: exposure. [2] Weight-for-height for gestational age category: 0: underweight, 1: normal, 2: overweight/obese. Low plasma volume: <2 L in the 1st, <2.6 L in the 2nd, and <2.8 L in the 3rd trimester. * Variable has been winsorized.

Table S2. Additional logistic regression models for Anemia: (A) Including serum iron instead of ferritin. (B) Including hepcidin instead of ferritin. (C) Including sTfR instead of ferritin.

**(A) Anemia (Hb <110 g/L)**	**OR**	***p***	**95% CI**	**Standardized Domin. Stat.**	**Ranking**
Serum iron, µmol/L	0.920	0.006	0.87, 0.98	0.29	1
[1] Weight-for-height category	0.435	0.005	0.24, 0.78	0.19	2
Parity	1.157	0.019	1.02, 1.31	0.14	3
Folic acid, nmol/L	0.963	0.118	0.92, 1.01	0.13	4
Vitamin A, µmoll/L	0.376	0.027	0.16, 0.89	0.12	5
[2] Wood smoke exposure	2.773	0.171	0.64, 11.95	0.07	6
[3]Low plasma volume	0.228	0.076	0.04, 1.16	0.06	7
Trimester	0.987	0.962	0.58, 1.68	0.01	8
Constant	41.873	0.007	2.81, 624.15		
**(B) Anemia (Hb <110 g/L)**	**OR**	***p***	**95% CI**	**Standardized Domin. Stat.**	**Ranking**
[1] Weight-for-height category	0.43	0.004	0.24, 0.76	0.23	1
Folic acid, nmol/L	0.95	0.031	0.91, 0.99	0.19	2
Parity	1.17	0.01	1.04, 1.33	0.18	3
Vitamin A, µmoll/L	0.36	0.021	0.15, 0.86	0.15	4
[2] Wood smoke exposure	3.31	0.104	0.78, 14.07	0.11	5
[3] Low plasma volume	0.21	0.065	0.04, 1.10	0.08	6
Hepcidin, µg/L	0.97	0.097	0.93, 1.01	0.06	7
Trimester	0.86	0.592	0.50, 1.48	0.01	8
Constant	41.30	0.009	2.52, 676.36		
**(C) Anemia (Hb <110 g/L)**	**OR**	***p***	**95% CI**	**Standardized Domin. Stat.**	**Ranking**
[1] Weight-for-height category	0.47	0.008	0.26, 0.82	0.21	1
Folic acid, nmol/L	0.95	0.031	0.91, 0.99	0.19	2
Parity	1.17	0.011	1.04, 1.32	0.18	3
Vitamin A, µmoll/L	0.39	0.029	0.17, 0.91	0.15	4
[2] Wood smoke exposure	3.19	0.121	0.74, 13.86	0.10	5
sTfR, mg/L	1.07	0.171	0.97, 1.17	0.08	6
[3] Low plasma volume	0.24	0.084	0.05, 1.22	0.07	7
Trimester	0.90	0.709	0.53, 1.53	0.01	8
Constant	12.78	0.061	0.89, 183.28		

(A) Model n = 210, overall fit statistic = 0.499, VIF = 1.13. (B) Model n = 210, overall fit statistics = 0.126, VIF = 1.15. (C) Model n = 210, overall fit statistics = 0.123, VIF = 1.14. [1] Weight-for-height for gestational age category: 0: underweight, 1: normal, 2: overweight/obese. [2] Wood smoke exposure categorized as 0: no exposure, 1: exposure. [3] Low plasma volume: <2 L in the 1st, <2.6 L in the 2nd, and <2.8 L in the 3rd trimester.
